# Supplementary material for: CD30 Lateral Flow and Enzyme-Linked Immunosorbent Assays for Detection of BIA-ALCL: A Pilot Study
Source: Cancers (Basel). 2023 Oct 25;15(21):5128. doi: 10.3390/cancers15215128 (PMC10649192; doi:10.3390/cancers15215128)
Supplement: Supplementary file 1 [file cancers-15-05128-s001.zip › cancers-2642467-supplementary.pdf]

Zyel et al.
Supplementary Methods

LFA assay

A lateral flow assay (LFA) was used to detect CD30 using LFA strips and detection particles manufactured in-house. LFA strips were made by striping mouse anti-CD30 antibody (R&D systems; cat# MAB291) on nitrocellulose membrane using a BioDot dispenser (BioDot; cat# XYZ30600124). Detection particles were made by conjugating red latex particles (ThermoFisher; cat# DR104) with affinity-purified Polyclonal goat anti-CD30 (R&D Systems, cat# AF229). Seroma fluid was diluted to specified concentrations in LFA running buffer, mixed with detection particles, and pipetted onto the sample pad of CD30 LFA strips. After 5 minutes, each strip was washed with additional LFA running buffer. A positive result was a red test line whose intensity corresponded to the concentration of CD30. A control line ensured the flow of detection particles and confirmed a negative result. The detailed LFA procedure is provided below.

CD30 strip preparation

To manufacture LFA strips, CN95 membrane (Sartorius; cat# 1UN95ER100025NTB) was assembled on a 30-cm backing adhesive card (DCN Diagnostics; cat# MIBA-020) along with an absorbent pad (Ahlstrom; cat# 440) and a sample pad (Ahlstrom; cat# 8980). The assembled membrane was striped using a BioDot dispenser (BioDot; cat# XYZ30600124), at a flow rate of 1 µL/cm and a dispensed volume of 30 µL per 30 cm card. The test line contained mouse anti-CD30 antibody (R&D systems; cat# MAB291) diluted to a concentration of 1 mg/mL in 1× PBS and 0.2% sucrose. The control line contained donkey polyclonal anti-goat antibodies (Jackson ImmunoResearch Laboratories Inc.; cat# 705-005-147) diluted to a concentration of 0.75 mg/mL in 1× PBS and 0.2% sucrose. Striped membranes were dried in a Robbins Scientific Micro Hybridization Incubator 2000 at 37°C for 30 min, then stored overnight at 20°C in a desiccator chamber (Totech; SuperDry Desiccant Cabinet; cat cat# SD-151-21) at 5% humidity. The striped card was cut into 3 mm strips using a KinBio ZQ2000 Guillotine Cutter and stored at 20°C in sealed 50 mL conical tubes (USA Scientific; cat# 5622-7261) with desiccant packs (Interteck Packaging; cat# IN1G51).

Conjugation of latex particles with CD30 antibody

Carboxylate-modified red latex particles (ThermoFisher; cat# DR104) were functionalized with affinity-purified Polyclonal goat anti-CD30 (R&D Systems, cat# AF229) using standard EDC-NHS chemical activation. Briefly, 100 µL particles at 0.5% solids were centrifuged (10 min at 16,500 × g) and washed twice with 50 mM MES buffer, pH 5.8. The particles were resuspended in 95 µL MES buffer and sonicated until no visible aggregates remained. Particles were activated by EDC (ThermoFisher; cat# A35391) and NHS (Millipore Sigma; cat# 130672) at a molar ratio of NHS:carboxyl groups of 20 (1.11 µL of 50 mg/mL NHS) and EDC:carboxyl groups of 2.5 (1.2 µL of 10 mg/mL EDC). NHS and EDC were added to the resuspended particle mixture sequentially (NHS followed by EDC) and placed on a benchtop rotator for 30 min at 20°C. After activation, the particles were washed twice by centrifugation in 1× PBS and resuspended by sonication. 25 µg of antibody (25 µL of 1 mg/mL suspended in 1× PBS) was added to the particles and incubated at 20°C on a benchtop rotator for 2 h. The mixture of particles and antibody was centrifuged (10 min at 16,500 × g), the supernatant was removed, and particles were resuspended in 4% BSA in 1× PBS and incubated for 1 hr at 20°C on a benchtop rotator. The particles were washed three times by centrifugation with 1× PBS, 1% BSA solution, resuspended at 0.5% solids in 100 µL 1× PBS, 1% BSA, and stored at 4°C.

LFA running protocol for IL-10

30 µL of sample was prepared by mixing 6 µL of a concentrated LFA running buffer (5× PBS (pH 7.4), 2.5% BSA (Millipore Sigma; cat# A9418), 1.25% Tween-20 (Millipore Sigma; cat# P1379), 1.5% PEG 3000 (Millipore Sigma; cat# 81227) with 24 µL of diluted seroma fluid (diluted as specified with diH_2O). The sample was then pipetted onto the sample pad of the LFA strip. After 5 minutes, 2.5×10^9 reporter particles diluted in LFA running buffer (1× PBS (pH 7.4), 0.5% BSA, 0.25% Tween-20, and 0.3% PEG 3000)

(20 µL total volume) were pipetted onto the sample pad of the LFA strip. After 5 minutes, the strips were washed with an additional 20 µL of LFA running buffer. The strips were run for a total of 15 minutes before imaging with a smartphone camera (iPhone 12).
